# Small sharks, big problems: DNA analysis of small fins reveals trade regulation gaps and burgeoning trade in juvenile sharks

**DOI:** 10.1126/sciadv.adq6214

**Published:** 2024-10-16

**Authors:** Diego Cardeñosa, Elizabeth A. Babcock, Stanley K. Shea, Huarong Zhang, Kevin A. Feldheim, Stephan W. Gale, DeEtta Mills, Demian D. Chapman

**Affiliations:** ^1^ Department of Biological Sciences, Florida International University, North Miami, FL, USA.; ^2^ Rosenstiel School of Marine and Atmospheric Science, University of Miami, Miami, FL 33149, USA.; ^3^ BLOOM Association, Central, Hong Kong, Special Administrative Region of China.; ^4^ Kadoorie Farm and Botanic Garden, Tai Po, Hong Kong, Special Administrative Region of China.; ^5^ Pritzker Laboratory for Molecular Systematics and Evolution, The Field Museum, Chicago, IL 60605, USA.; ^6^ Center for Shark Research, Mote Marine Laboratory, Sarasota, FL 34236, USA.

## Abstract

Many shark species have been overexploited for international markets, including fins for shark fin soup in Southeast Asia. Previous studies highlighted the value of large, threatened shark species, regulated under CITES Appendix II. However, sampling biases may have overlooked small shark species. Here, we address this by identifying species from ~4000 small shark fins in Hong Kong. These fins included species not recorded in previous surveys, raising the market’s species diversity to 106. Nearly 75% of the small fins came from small shark species and 58.1% of small species were threatened with extinction. We identified an important CITES listing gap: Trade in 19 small, threatened species, especially from the family Triakidae, is unregulated. In addition, a quarter of small fins come from large sharks, indicating that substantial exploitation of juveniles is occurring and may be affecting fisheries sustainability. Enhanced surveillance of small shark fin trade is essential to ensure effective conservation under emerging trade regulations.

## Teaser

Survey uncovers rampant fin trade of small and juvenile sharks, exposing regulatory gaps.

## INTRODUCTION

Overfishing is the most immediate threat to sharks and rays globally (*1*, *2*), with international trade being an ultimate driver of unsustainable fishing practices (*3*). Large-bodied shark species tend to have life-history traits that make them more vulnerable to overexploitation than smaller species (*4*). Large species also produce larger volumes of meat per individual, and large shark fins fetch much higher prices than small fins in the markets of Southeast Asia, which encourages targeting of large shark species in many fisheries (*5*). Clarke *et al.* (*5*) assessed the species composition in wholesale auction shark fin markets in Hong Kong Special Administrative Region of the People’s Republic of China (hereafter referred to as Hong Kong) based on 11 valuable market categories, avoiding categories where mixed fins where simply sorted by size because of relatively low value (e.g., small fins). This effort resulted in the identification of 14 important species or species groups all of which were large-bodied cosmopolitan sharks (*5*). From 2013 onward, nearly all the large-bodied species that are prominent in the fin trade have been added to Appendix II of the Convention on International Trade of Endangered Species (CITES) to better regulate trade and promote conservation.

More recently, systematic shark fin market surveys conducted in Hong Kong (*3*, *6*, *7*) and Guangzhou, Mainland China (*8*), based on the monthly sampling of processed shark fin trimmings, have expanded our understanding of the species diversity at these global trade hubs. These fin trimmings are a by-product of the processing of shark fins, where processors cut the fins at the base and along the leading and trailing edges of the fins to make them look aesthetically appealing and remove unwanted parts. Similar to what was found by Clarke *et al.* (*5*), the markets are heavily skewed toward a small subset of large cosmopolitan species such as blue shark (*Prionace glauca*), silky shark (*Carcharhinus falciformis*), scalloped hammerhead (*Sphyrna lewini*), smooth hammerhead (*Sphyrna zygaena*), blacktip complex (i.e., *Carcharhinus limbatus*, *Carcharhinus leiodon*, *Carcharhinus tilstoni*, and *Carcharhinus amblyrhynchoides*), and bull shark (*Carcharhinus leucas*). However, this survey detected more than 86 species of chondrichthyans (sharks, rays, and chimeras) in the trade including 37 species of small-bodied sharks, which are defined here as having a size of maturity below 120 cm in total length (TL). These small species collectively represented 8.9% of all sampled trimmings (*3*). The fin trimming survey might oversample larger sharks given that (i) large fins are likely to yield more trimmings per fin, (ii) unwanted cartilage insertions at the base of large fins are more prominent, and (iii) trimming small fins risks compromising the integrity of the entire fin. Cardeñosa *et al.* (*9*) conducted shark fin inspections with the border control authorities in Hong Kong, where a container was found with a ~8000 kg of consignment entirely composed of small shark fins. Typically, these small fins are not thoroughly inspected due to the challenges with visual species identification (*10*), and up to now, there are no comprehensive assessments of the species being traded in small fins. Small fins from different sacks in the container were randomly sampled and genetically tested and found to be a mix of small coastal species, mostly milk shark (*Rhizoprionodon acutus*; 55%), and juveniles of large species [e.g., scalloped hammerhead shark; (*9*)]. Similarly, a preliminary systematic survey of small fins in the retail markets of Hong Kong (*N* = 475) revealed 29 different species, most of which represented small coastal species (62.9%) and juveniles of larger species [37.1%; (*11*)].

Here, we expand upon the initial findings presented by Cardeñosa *et al.* (*11*) by surveying 5635 processed small fins collected over a year (2019) in the retail markets of Hong Kong. In addition, we incorporate the latest data from long-term surveys spanning the period 2014 to 2021 (*n* = 20,424), focusing on shark fin trimmings in these markets. Our objectives were to (i) provide a better estimate of overall species diversity in the market, (ii) assess the extent to which threatened small species are traded and subject to international trade regulations, and (iii) identify large species that are common in the small fin trade, indicating that they are often caught and traded before reaching maturity.

## RESULTS

**Fig. 1.**
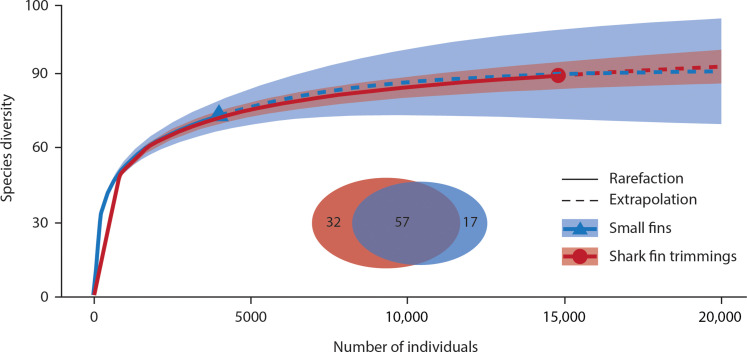
Rarefaction curves and extrapolation of the abundance data analysis. Species diversity estimates for both surveys (small fins and shark fin trimmings) with a projection of 20,000 individual samples. Numbers in Venn diagram represent the number of species shared and unique to each survey.

**Fig. 2.**
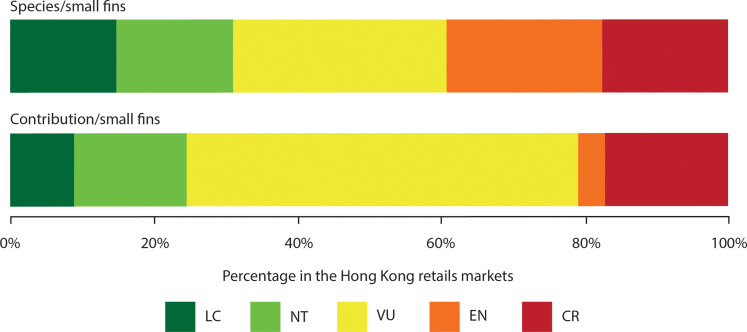
Relative contribution of IUCN categories to the small fin survey. Bar-plot showing the contribution of threatened [vulnerable (VU), endangered (EN), or critically endangered (CR)] species to the shark fin trade based on small fin data by number of species (**top**) and by contribution of fins (**bottom**). NT, near threatened; LC, least concern.

The combined survey of small fins (*N* = 3982 identified) and fin trimmings (*N* = 14,771 identified) revealed that at least 106 chondrichthyan species occur in the international fin trade. Seventy-four elasmobranch species or species complexes were detected in the small fin survey in 2019, while 272 samples were only identified to the genus level (Table 1). Similarly, from February 2014 to December 2021, 90 species or species complexes were identified in the trimmings, while a further 1163 samples were only identified to genus level (Table 1). The rarefaction curves and extrapolation of the abundance data analysis for both surveys (i.e., small fins versus fin trimmings) followed almost an identical trend and reached a plateau with a projection of 20,000 individual samples (Fig. 1). Fifty-seven species were detected in both surveys, with 18 and 32 species being unique to the small fin and fin trimming surveys, respectively (Fig. 1). We determined that, based on the combined species richness estimates and their confidence intervals, a maximum of 60 taxa in the small fin market (Table 2) and 54 taxa in the fin trimming retail market (Table 3) could be present. Of the 74 species found in the small fin survey, 51 (68.9%) were assessed as being threatened with extinction by the International Union for Conservation of Nature (IUCN) Red List (Fig. 2A). Moreover, 75.5% of all small fins sampled were from species in threatened categories (Fig. 2B).

**Table 1.** Species composition and contribution to the Hong Kong markets.Species and species groups found in the small fin survey (*N* = 4254) and an updated species list from the fin trimming survey in the retail markets of Hong Kong (*N* = 15,934) with their respective counts and % in each survey, IUCN status, CITES listing, presence in FAO-MFAs, habitat, and population trend. Habitat and populations trends are based on the IUCN Red List assessments for each species. N/A, not applicable.

Species	Count SmF^*^	% SmF	Count FiT^†^	% FiT	IUCN	CITES-listed	FAO-MFAs^‡^	Habitat	Trend^§^
*R. acutus*	919	21.6	341	2.14	VU	Yes	7	Small coastal	↓
*M. punctulatus*	364	8.56	118	0.74	VU	No	2	Small coastal	↓
*M. antarcticus*	288	6.77	0	0	LC	No	2	Small coastal	→
*C. punctatum*	259	6.09	59	0.37	NT	No	3	Small coastal	↓
*M. schmitti*	255	5.99	77	0.48	CR	No	1	Small coastal	↓
*S. lewini*	253	5.95	831	5.21	CR	Yes	11	Large pelagic	↓
*Carcharhinus sorrah*	205	4.82	243	1.52	NT	Yes	4	Small coastal	↓
*Rhizoprionodon porosus/terraenovae*	203	4.77	42	0.26	VU	Yes	2	Small coastal	↓
Blacktip complex	172	4.04	877	5.49	VU	Yes	13	Large coastal	↓
*S. zygaena*	153	3.6	544	3.41	VU	Yes	14	Large pelagic	↓
*C. falciformis*	99	2.33	1992	12.48	VU	Yes	12	Large pelagic	↓
*G. galeus*	84	1.97	59	0.37	CR	No	10	Small coastal	↓
*C. brevipinna*	66	1.55	286	1.79	VU	Yes	10	Large coastal	↓
*C. porosus*	60	1.41	16	0.1	CR	Yes	4	Small coastal	↓
*Rhizoprionodon oligolinx*	51	1.20	15	0.09	NT	Yes	4	Small coastal	↓
*Carcharhinus sealei/coatesi*	51	1.20	3	0.02	VU	Yes	2	Small coastal	↓
*Carcharhinus tjutjot*	46	1.08	1	0.01	VU	Yes	2	Small coastal	↓
S*. tiburo*^¶^	43	1.01	6	0.04	EN	Yes	5	Small coastal	↓
*Carcharhinus acronotus*	43	1.01	21	0.13	EN	Yes	2	Small coastal	↓
*Rhizoprionodon taylori*	29	0.68	41	0.26	LC	Yes	2	Small coastal	?
*Loxodon macrorhinus*	27	0.63	15	0.09	NT	Yes	5	Small coastal	↓
*C. leucas*	25	0.59	286	1.79	VU	Yes	12	Large coastal	↓
*Carcharhinus galapagensis/obscurus*	19	0.45	90	0.56	EN	Yes	15	Large pelagic	↓
*Hemigaleus microstoma*	18	0.42	3	0.02	VU	No	4	Small coastal	↓
*Carcharhinus macloti*	18	0.42	7	0.0.04	NT	Yes	4	Small coastal	↓
*Carcharhinus amboinensis*	17	0.40	255	1.60	VU	Yes	5	Large coastal	↓
*Carcharhinus isodon*	17	0.40	8	0.05	NT	Yes	4	Large coastal	→
*Squatina californica*	17	0.40	1	0.01	NT	No	1	Small coastal	↓
*C. dussumieri*	14	0.33	29	0.18	EN	Yes	1	Small coastal	↓
*P. glauca*	11	0.26	6405	40.13	NT	Yes	15	Large pelagic	↓
*Galeocerdo cuvier*	11	0.26	110	0.69	NT	No	15	Large pelagic	↓
*Hemigaleus australiensis*	11	0.26	8	0.05	LC	No	2	Small coastal	?
*Sphyrna mokarran*	9	0.21	169	1.06	CR	Yes	14	Large coastal	↓
*Hemipristis elongata*	9	0.21	28	0.18	VU	No	4	Small coastal	↓
*Mustelus henlei*	9	0.21	27	0.17	LC	No	2	Small coastal	?
*Carcharhinus melanopterus*	8	0.19	16	0.1	VU	Yes	4	Small coastal	↓
*Carcharhinus amblyrhynchos*	7	0.16	73	0.46	EN	Yes	4	Large coastal	↓
*Eusphyra blochii*	7	0.16	5	0.03	EN	Yes	4	Large coastal	↓
*Triaenodon obesus*	6	0.14	2	0.01	VU	Yes	5	Small coastal	↓
*Rhynchobatus australiae*	6	0.14	77	0.48	CR	Yes	4	Large coastal	↓
*Mustelus mosis*	6	0.14	15	0.09	NT	No	2	Small coastal	↓
*Mustelus stevensi*	6	0.14	0	0	LC	No	1	Small coastal	↓
*Isurus oxyrinchus*	5	0.12	353	2.21	EN	Yes	15	Large pelagic	↓
*Sphyrna tudes*	5	0.12	5	0.03	CR	Yes	2	Small coastal	↓
*Furgaleus macki*	5	0.12	0	0	LC	No	1	Small coastal	↑
*Notorynchus cepedianus*	4	0.09	0	0	VU	No	8	Large coastal	↓
*Carcharhinus fitzroyensis*	3	0.07	0	0	LC	Yes	2	Small coastal	?
*Rhynchobatus springeri*	3	0.07	8	0.05	CR	Yes	2	Small coastal	↓
*Hemitriakis falcata*	3	0.07	1	0.01	LC	No	1	Small coastal	?
*Squatina armata*	3	0.07	0	0	CR	No	1	Small coastal	↓
*Carcharhinus brachyurus*	2	0.05	31	0.19	VU	Yes	10	Large coastal	↓
*Chaenogaleus macrostoma*	2	0.05	0	0	VU	No	4	Small coastal	↓
*Mustelus canis*	2	0.05	150	0.94	NT	No	3	Small coastal	↓
*Glyphis garricki*	2	0.05	0	0	CR	Yes	2	Large coastal	↓
*Carcharhinus perezi*	2	0.05	0	0	EN	Yes	2	Large coastal	↓
*Glyphis gangeticus*	2	0.05	0	0	CR	Yes	2	Large coastal	↓
*Iago omanensis*	2	0.05	4	0.03	LC	No	1	Small coastal	→
*Carcharhinus altimus/plumbeus*	2	0.05	219	1.37	EN	Yes	14	Large coastal	↓
*Alopias superciliosus*	1	0.02	25	0.16	EN	Yes	14	Large pelagic	↓
*Lamna nasus*	1	0.02	9	0.06	VU	Yes	11	Large pelagic	↓
*Alopias pelagicus*	1	0.02	175	1.10	EN	Yes	6	Large pelagic	↓
*Mustelus*	1	0.02	18	0.11	EN	No	4	Small coastal	↓
*Mustelus asterias*	1	0.02	0	0	NT	No	3	Small coastal	↓
*Rhynchobatus laevis*	1	0.02	31	0.19	CR	Yes	3	Large coastal	↓
*Paragaleus randalli*	1	0.02	0	0	VU	No	3	Small coastal	↓
*Acroteriobatus variegatus*	1	0.02	0	0	CR	No	2	Small coastal	↓
*Mustelus manazo*	1	0.02	0	0	EN	No	2	Small coastal	↓
*Leptocharias smithii*	1	0.02	0	0	VU	No	2	Small coastal	↓
*Mustelus lenticulatus*	1	0.02	0	0	LC	No	1	Small coastal	↓
*Lamiopsis tephrodes*	1	0.02	0	0	EN	No	1	Large coastal	↓
*Hydrolagus novaezealandiae*	1	0.02	13	0.08	LC	No	1	Small coastal	→
*Glyphis*	1	0.02	3	0.02	VU	Yes	1	Small coastal	↓
*Hemitriakis japanica*	1	0.02	0	0	EN	No	1	Small coastal	↓
*Carcharhinus longimanus*	0	0	118	0.74	CR	Yes	12	Large pelagic	↓
*Lamna ditropis*	0	0	88	0.55	LC	Yes	3	Large pelagic	→
*Dalatias licha*	0	0	72	0.45	VU	No	11	Large Deep	↓
*Negaprion acutidens*	0	0	43	0.27	EN	Yes	4	Large coastal	↓
*Scoliodon laticaudus*	0	0	35	0.22	NT	Yes	4	Small coastal	↓
*Squalus acanthias*	0	0	35	0.22	VU	No	11	Small coastal	↓
*Glaucostegus cemiculus*	0	0	22	0.14	CR	Yes	3	Large coastal	↓
*M. mosis*	0	0	15	0.09	NT	No	2	Small coastal	↓
*Negaprion brevirostris*	0	0	10	0.06	VU	Yes	7	Large coastal	↓
*Centroscymnus coelolepis*	0	0	8	0.05	NT	No	11	Small deep	↓
*Rhizoprionodon longurio*	0	0	7	0.04	VU	Yes	2	Large coastal	↓
*Chiloscyllium plagiosum*	0	0	7	0.04	NT	No	4	Small coastal	↓
*Rhynchobatus djiddensis*	0	0	7	0.04	CR	Yes	1	Large coastal	↓
*Mustelus griseus*	0	0	7	0.04	NT	No	2	Small coastal	↓
*Lamiopsis temminckii*	0	0	6	0.04	EN	Yes	3	Large coastal	↓
*Isurus paucus*	0	0	5	0.03	EN	Yes	14	Large pelagic	↓
*Deania profundorum*	0	0	4	0.03	NT	No	4	Small deep	↓
*Mustelus lunulatus*	0	0	3	0.02	LC	No	1	Small coastal	?
*Alopias vulpinus*	0	0	3	0.02	VU	Yes	15	Large pelagic	↓
*Centrophorus squamosus*	0	0	3	0.02	EN	No	7	Small deep	↓
*Rhinobatos schlegelii*	0	0	3	0.02	CR	Yes	1	Small coastal	↓
*Centrophorus isodon*	0	0	2	0.01	EN	No	4	Small deep	↓
*Rhina ancylostoma*	0	0	2	0.01	CR	Yes	4	Large coastal	↓
*Chiloscyllium hasseltii*	0	0	2	0.01	EN	No	2	Small coastal	↓
*Callorhinchus callorynchus*	0	0	1	0.01	VU	No	2	Small deep	↓
*Carcharias taurus*	0	0	1	0.01	CR	No	11	Large coastal	↓
*Hexanchus griseus*	0	0	1	0.01	NT	No	15	Large deep	↓
*Stegostoma tigrinum*	0	0	1	0.01	EN	No	4	Large coastal	↓
*Glaucostegus granulatus*	0	0	1	0.01	CR	Yes	2	Large coastal	↓
*Rhinobatos hynnicephalus*	0	0	1	0.01	EN	Yes	2	Small coastal	↓
*Sphyrna corona*	0	0	1	0.01	CR	Yes	2	Small coastal	↓
*Mustelus sinusmexicanus*	0	0	1	0.01	LC	No	1	Small coastal	↑
*Carcharhinus* sp.	143	3.36	643	4.03	N/A	Yes	N/A	N/A	N/A
*Callorhinchus* sp.	59	1.39	214	1.34	N/A	No	N/A	N/A	N/A
*Mustelus* sp.	54	1.27	90	0.56	N/A	No	N/A	N/A	N/A
*Squatina* sp.	12	0.28	9	0.06	N/A	No	N/A	N/A	N/A
*Rhizoprionodon* sp.	2	0.05	33	0.21	N/A	Yes	N/A	N/A	N/A
*Rhynchobatus* sp.	1	0.02	42	0.26	N/A	Yes	N/A	N/A	N/A
*Orectolubus* sp.	1	0.02	0	0	N/A	No	N/A	N/A	N/A
*Alopias* sp.	0	0	38	0.24	N/A	Yes	N/A	N/A	N/A
*Centrophorus* sp.	0	0	33	0.21	N/A	No	N/A	N/A	N/A
*Chiloscyllium* sp.	0	0	20	0.13	N/A	No	N/A	N/A	N/A
*Glyphis* sp.	0	0	8	0.05	N/A	Yes	N/A	N/A	N/A
*Hydrolagus* sp.	0	0	7	0.04	N/A	No	N/A	N/A	N/A
*Squalus* sp.	0	0	5	0.03	N/A	No	N/A	N/A	N/A
*Sphyrna* sp.	0	0	5	0.03	N/A	Yes	N/A	N/A	N/A
*Lamna* sp.	0	0	3	0.03	N/A	Yes	N/A	N/A	N/A
*Loxodon* sp.	0	0	3	0.02	N/A	Yes	N/A	N/A	N/A
*Iago* sp.	0	0	2	0.01	N/A	No	N/A	N/A	N/A
*Glaucostegus* sp.	0	0	1	0.01	N/A	Yes	N/A	N/A	N/A
*Pristis* sp.	0	0	1	0.01	N/A	Yes	N/A	N/A	N/A
*Deania* sp.	0	0	1	0.01	N/A	No	N/A	N/A	N/A

*Counts for the small fin survey.

†Counts for the fin trimming survey.

‡Number of FAO-MFAs where distribution range overlaps.

§Population trend based on IUCN Red List: ↓, decreasing; →, stable; ↑, increasing; ?, unknown.

¶Denotes the species complex comprising *Sphyrna tiburo* and *Sphyrna alleni*.

**Table 2.** Species richness estimations for small fins using abundance data. CI, confidence interval; SE, standard error; MLE, maximum likelihood estimation; ACE, abundance-based coverage estimator.

Model	Estimate	SE	95% CI
Homogeneous model	77.559	3.132	73.987–87.556
Homogeneous (MLE)	72.000	2.182	75.113–84.600
Chao1 (*35*)	90.281	12.230	77.550–132.212
Chao1-bc	86.996	9.981	76.576–121.142
iChao (*36*)	92.281	14.380	77.761–112.137
ACE (*37*)	87.206	8.016	77.761–112.137
ACE-1 (*37*)	92.968	12.443	79.146–133.531
First-order jackknife	87.996	5.656	80.163–103.344
Second-order jackknife	96.993	9.795	83.915–124.427

**Table 3.** Species richness estimations for shark fin trimmings using abundance data. CI, confidence interval; SE, standard error; MLE, maximum likelihood estimation; ACE, abundance-based coverage estimator.

Model	Estimate	SE	95% CI
Homogeneous Model	92.316	2.223	90.004–99.946
Homogeneous (MLE)	89.000	1.942	90.814–98.928
Chao1 (*35*)	103.399	11.179	92.747–144.331
Chao1-bc	99.999	8.478	91.885–130.940
iChao (*36*)	106.243	7.000	97.020–126.074
ACE (*37*)	96.751	4.750	91.563–112.444
ACE-1 (*37*)	98.453	6.185	91.934–119.455
First-order jackknife	100.999	4.899	94.558–114.903
Second-order jackknife	107.999	8.485	97.236–132.824

**Fig. 3.**
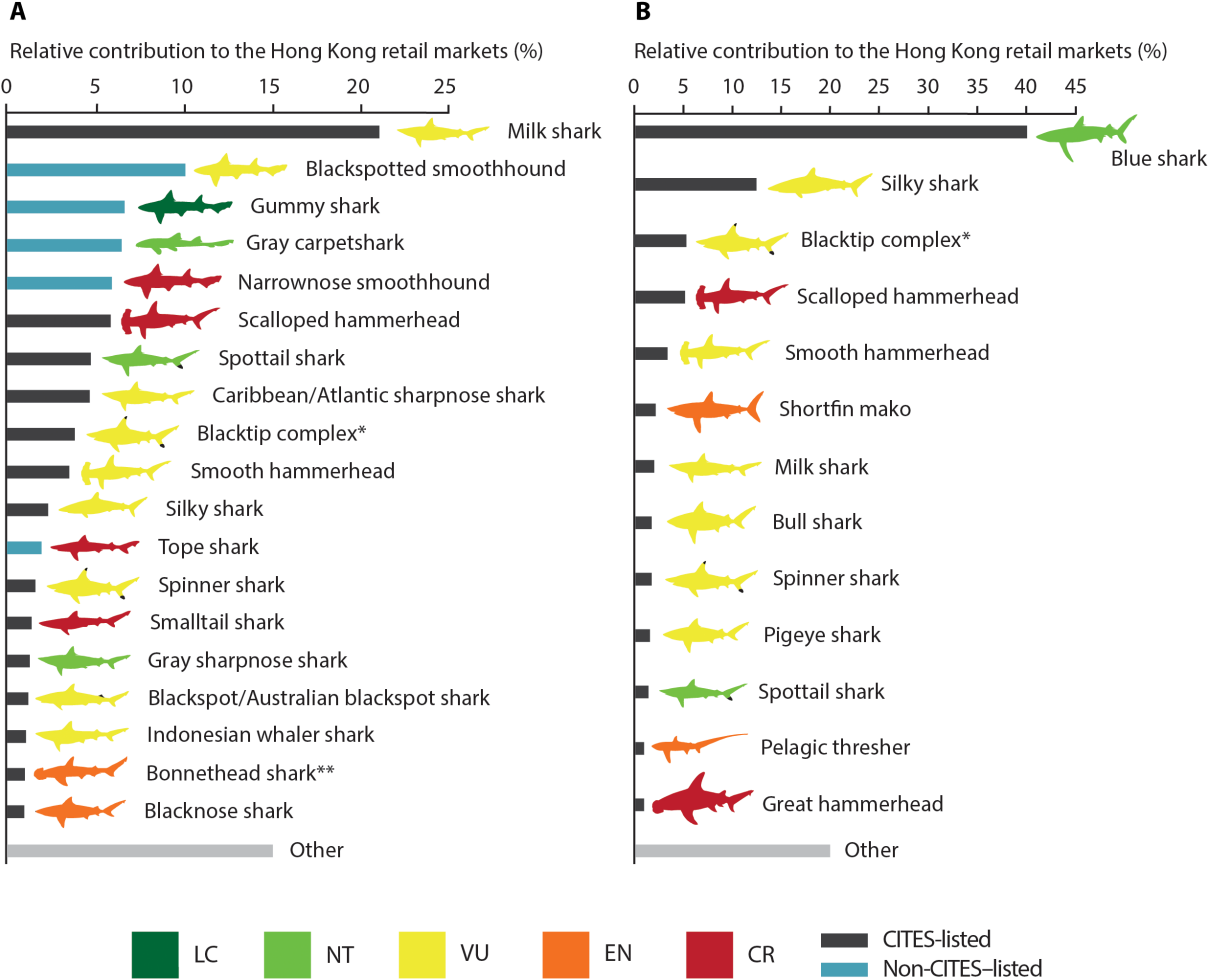
Main species composition of the Hong Kong shark fin markets. Bar plots showing the relative contribution of the most prevalent species to the small shark fins (**A**) and the shark fin trimmings (**B**) trade, as sampled in Hong Kong. Only species contributing >1% to the markets were included. All species are color-coded to depict their IUCN Red List status. Asterisk (*) denotes the species complex comprising *C. limbatus*, *C. amblyrhynchoides*, *C. leiodon*, and *C. tilstoni.*

The dominant species was the milk shark (*R. acutus*), which was also the dominant small species detected in fin trimmings. When combining the surveys, we detected 62 small species in the fin trade, 36 (58.1%) of which are threatened, but only 23 (37.1%) are listed on CITES Appendix II. In contrast, among the 44 large species found in the fin trade, 37 (83.7%) are threatened, and 37 (83.7%)—although not the same 37 species—are listed on CITES Appendix II. Some of the small threatened species that are not listed on CITES were highly traded in the small fin category including black-spotted smooth hounds (*Mustelus punctulatus*), narrownose smoothhounds (*Mustelus schmitti*), and tope sharks (*Galeorhinus galeus*; Fig. 3A), all of which are in the family Triakidae.

Large coastal sharks that use coastal nursery areas accounted for 25.7% of the identified species and contributed a combined 18% of the tested fins. Notably, the critically endangered scalloped hammerhead was the sixth most common species overall (5.95% of all fins; Fig. 3A). Other prominent large coastal species commonly found in the small fin survey were the blacktip complex (4.04% of all fins), smooth hammerhead (3.6% of all fins), and spinner shark (*Carcharhinus brevipinna*; 1.55% of all fins). Some pelagic species not typically found in coastal waters were found in the small fin survey, especially the silky shark (2.33% of all fins)*.*

**Fig. 4.**
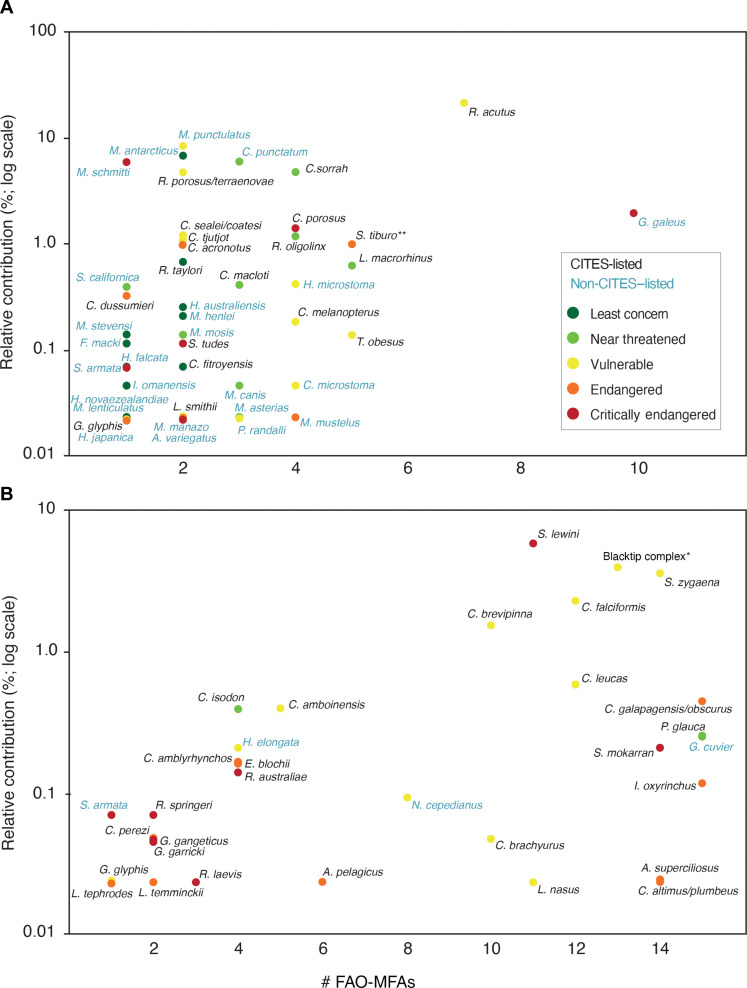
Relative contribution of species found on the small fin survey and their geographic distribution. Plot of observed relative contribution in the Hong Kong small shark fin retail markets by species and their geographic distribution for small species (**A**) and for large species (**B**). Asterisk (*) denotes the species complex comprising *C. limbatus*, *C. amblyrhynchoides*, *C. leiodon*, and C. tilstoni. ** denotes the species complex comprising *Sphyrna tiburo* and *Sphyrna alleni*.

**Fig. 5.**
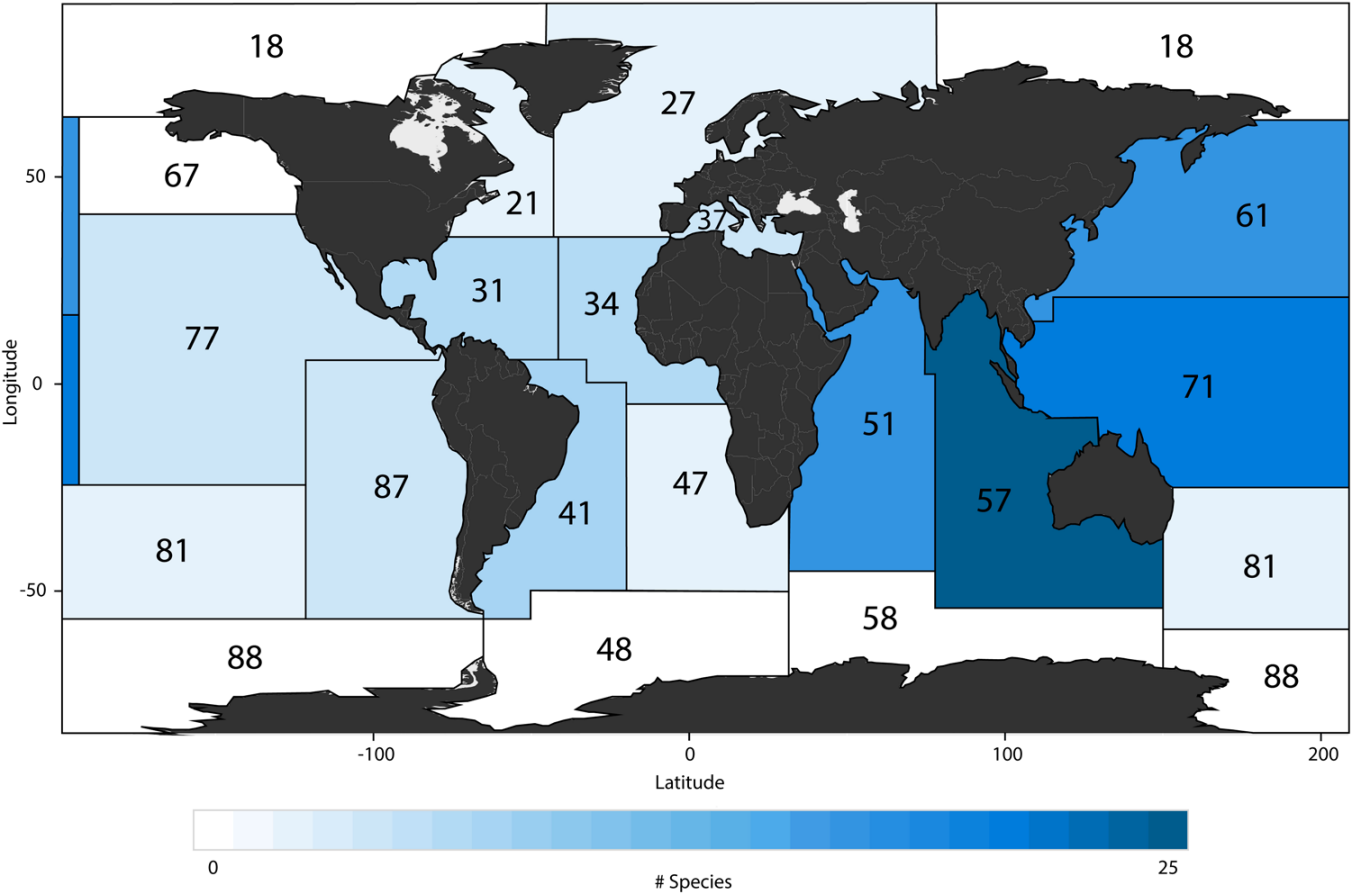
Geographic distribution of species present in the small fin trade. Overlapping species distributions present in the small fin trade to FAO-MFAs. Numbers denote area codes assigned by FAO.

Small and large species’ relative contribution in the small fin category was positively correlated with their geographic range size (Kendall correlation = 0.35, *P* = 0.01; Spearman correlation = 0.44, *P* = 0.01): range-restricted species had a lower contribution in the small shark fin trade than wider ranging species (Fig. 4). When we mapped the distributions of the range-restricted species [i.e., species with a distribution of seven or less Food and Agriculture Organization Major Fishing Areas (FAO-MFAs)] identified in the small shark fin survey, we found that the Western Pacific region, particularly the Coral Triangle, stood out as the area where most of the identified species naturally occur (Fig. 5).

## DISCUSSION

This study provides the most comprehensive assessment of the species diversity of one of the world’s largest shark fin markets, overcoming one limitation of previous surveys—the emphasis on large species—by combining DNA analysis of both small whole shark fins and shark fin trimmings taken from large and small fins. Previous studies based on auction records or fin trimmings, the latter being a by-product of processing large and small fins but likely being proportional to fin size (*3*, *6*, *7*), were dominated by large species with five species/complexes comprising more than 67% of the sampled trimmings. In contrast, small fins were dominated by small species with nine species making up 67% of the total relative contribution. Combining these surveys resulted in the identification of at least 106 species overall, and species richness estimations suggest that up to 60 more species could be found with additional sampling. Our combined survey therefore indicates that at least 18.5% of all shark species are traded for their fins, regardless of their body size. Similarly to Cardeñosa *et al.* (*3*), our results indicated that two-thirds (68.9%) of the species found in the small shark fins collected in the Hong Kong retail markets were threatened with extinction. This proportion increased to 75.5% when examining the total contribution of IUCN threatened categories to the total number of samples identified.

The combined survey highlighted some important gaps in our understanding of the threat of international trade to small sharks and the application of international trade regulations to address this issue. As expected, we found that large species in the fin trade were generally more threatened than small species, which is consistent with their relative productivity and their higher value per individual. However, we still found that most small species in the fin trade are threatened with extinction (~58% of species, collectively representing 50.8% of all the small fins in trade). The few species that are not threatened either primarily occur in jurisdictions with strong fisheries governance (e.g., gummy shark, *Mustelus antarcticus*) or are near threatened and declining, indicating that management interventions are likely needed to prevent them becoming threatened (e.g., gray carpet shark, *Chiloscyllium punctatum*). CITES listings for sharks have historically focused on large species, but recently many small species were listed when the entire family Carcharhinidae and family Sphyrnidae were included in the Appendix II. Yet, our survey highlights an important CITES listing gap: There are at least 19 species of small, threatened shark species in the international fin trade that are not regulated by CITES.Around a third of the species found in the small fin survey are in the family Triakidae, and this family represented 30.5% of all sampled fins. The vulnerable and critically endangered blackspotted smoothhound (*M. punctulatus*; VU), narrownose smoothhound (*M. schmitti*; CR), and tope shark (*G. galeus*; CR) ranked the second, fifth, and 14th most common species in the small shark fin survey overall, respectively. Many of the small, threatened sharks might have particularly high extinction risk given their restricted geographical distributions, which reduces the likelihood of them having any populations in areas where fishing is absent or well regulated. These findings all support more consideration of small, threatened species in the fin trade for CITES listing especially the highly traded family Triakidae.

The majority of the species found in the small shark fin category occur in tropical and subtropical areas, mostly outside high-capacity nations (e.g., United States, Canada, Australia, and New Zealand); where implementation of shark fisheries management is challenging, where capacity building to enforce international trade regulations needs to be prioritized (*12*); and where illegal, unreported, and unregulated fisheries occur (*13*–*15*). Several recent elasmobranch extinctions have been reported in coastal fisheries in heavily fished tropical regions, outside high-capacity nations (*13*, *15*). Many species with high relative contributions in the small shark fin trade had similar extinction risk profiles such as the black-spotted smooth hounds, narrownose smoothhounds, whitecheek shark (*Carcharhinus dussumieri*; EN), small tail shark (*Carcharhinus porosus*; CR), and the tope shark (*G. galeus*; CR). Without strong management actions and implementation, these species could face a more immediate extinction risk than wider ranging species. While there is a strong correlation between relative contribution and geographical range for small species (Fig. 4A), at least 13 threatened small species with a relative contribution higher than 0.1% occur in four or fewer FAO-MFAs. The common presence of range-restricted species in a large trade, involving thousands of tons of fins per year (*7*), suggests heavy fishing pressure in these areas and underscores the need for better fisheries management and trade regulations enforcement to protect these threatened species.

Most of the species found in the small shark fin survey had overlapping distribution ranges in the Western Pacific and Eastern Indian oceans (Fig. 5). These regions are a global marine biodiversity hotspot with many species occurring in the coral triangle (*16*), including elasmobranchs (*17*), many of which were found in our survey. Species in these regions have high levels of endemism and are subject to a wide range of fishing pressures, including those in the largest shark catching nation by volume [i.e., Indonesia; (*18*)], and are subject to patchy conservation (e.g., Marine Protected Areas) and fisheries management [e.g., shark fishing quotas (Australia); (*19*)]. However, other species present in our small fin survey are endemic to certain regions with little or nothing in place in terms of conservation and fisheries management plans, highlighting their elevated extinction risk profile. This is the case with the blackspotted (VU) and narrownose smooth hounds (CR) that are endemic to the Mediterranean Sea and Northern Africa, as well as the Southwestern Atlantic, respectively, both of which are regions with high fishing pressures with limited capacity for fisheries management and sustainability (*20*, *21*).

Cardeñosa *et al.* (*11*) assessed that the majority (91.8%) of small fins in the Hong Kong markets were primary fins (i.e., first dorsal, pectoral fins, and lower lobe of caudal fin); therefore, we assume that small fins from large-bodied species found in this fin category are mainly from juvenile sharks. We also found a correlation between relative contribution and geographical range for large species in the small fin survey. The most common large species in the small fin survey are the scalloped and smooth hammerheads, blacktip, spinner, and bull sharks (Fig. 4B). All these species are tropical, cosmopolitan, threatened, and use coastal areas as nurseries where aggregating juveniles are frequently caught by longline, gillnet, and trawl fisheries (*22*–*24*).

Although some demographic models suggest that an overemphasis on reducing juvenile mortality is not the best strategy to achieve shark conservation (*25*), our findings suggest that many of the species found prominently in both trimmings and small fins are likely being fished across a wide range of sizes and often below size at first maturity, which can contribute to increased extinction risk (*26*). The trade in small fins is partially reliant on small, productive shark species (e.g., *Rhizoprionodon* and *Mustelus* spp.) and partially reliant on juveniles of large, less productive species (e.g., *Sphyrna* and *Carcharhinus* spp.), and regardless of size and productivity, most of these species are threatened with extinction and listed on CITES Appendix II. Our results further highlight more small sharks (e.g., family Triakidae) may qualify for CITES Appendix II listings in the future. We therefore suggest that consignments of small fins must not be overlooked and be subject to thorough inspection by CITES parties and authorities. Given the large and potentially growing number of listed small sharks and the prevalence of juveniles of listed species in the fin trade, it is implausible that any consignment of small fins would not require accompanying CITES export permits since 2024. Unfortunately, existing morphological guides for CITES-listed sharks fins suggest that they should only be used for fins larger than 10 cm at the base to avoid possible misidentification with very small specimens and unlisted small coastal species (*10*). Therefore, in-port molecular testing could be used more widely to detect illicit trade in CITES-listed species in small fins (*9*, *21*, *27*), especially consignments originating from countries/territories catching and trading large volumes of these small coastal species, (e.g., Western Pacific and Indian Oceans). In addition, increased inspection and genetic capacity building and resources are needed in exporting and importing countries/territories worldwide (*12*) because current capacity is likely to have already been exceeded by this high-volume trade (*7*). Collaborative initiatives, supported by international bodies and stakeholders in developed countries/territories, should be implemented in developing nations to transfer knowledge and relevant technologies and assist these nations to meet the challenge of improving long-term trade sustainability and enhancing the conservation of sharks (*12*, *28*).

## MATERIALS AND METHODS

### Sample collection in retail markets

Sampling of small fins (i.e., fin base less than 10 cm) was conducted between December 2018 and December 2019. Every month, one bag (~0.6 kg) was purchased from five randomly selected vendors from a list of >300 retail vendors in the Sheung Wan and Sai Ying Pun Districts of Hong Kong. A total of 95 fins were randomly selected from each bag for genetic identification. Sampling of shark fin trimmings followed a similar approach. Between February 2014 and January 2015, 10 vendors were randomly selected every 2 weeks, and two bags of trimmings were purchased. From February 2015 to December 2021, this process continued monthly. Ten random trimmings were selected from each bag for genetic identification.

### DNA extractions and mini barcoding

The genetic identification of selected samples was achieved following the protocols by Cardeñosa *et al.* (*29*). Genomic DNA was extracted by cutting a small portion of each fin (~2 mm^2^) and adding it to a polymerase chain reaction tube with 200 μl of 10% Chelex Resin (Bio-Rad, Hercules, CA, USA). Samples were incubated for 20 min at 60°C and for 25 min at 99°C, followed by a brief centrifugation and storage at 4°C (*29*). A mini barcoding assay was then used to amplify two portions of the mitochondrial cytochrome oxidase I [150 and 200 base pairs (bp)] following the thermal conditions and sequencing protocols by Cardeñosa *et al.* (*29*). All forward and reverse sequences underwent visual inspection, and priming sites were removed using Geneious Pro version 3.6.1 (accessible at www.geneious.com). These trimmed sequences were then cross-referenced with the BOLD (FISH-BOL) and BLAST (GenBank) online databases to identify them at the lowest taxonomic level feasible (e.g., genus and/or species). Samples were considered identifiable to the species level when the closest match in BLAST showed at least 2-bp differences to our target sequence or when BOLD returned a 100% species level match.

### Data analysis

After genetic identification, a rarefaction curve was constructed using iNEXT Online (*30*) to estimate the overall species count (i.e., species diversity) at specific sample sizes (i.e., abundance data) for both surveys (i.e., small fins versus fin trimmings). This estimation was based on the unified rarefaction and extrapolation sampling curves of Hill numbers for *q* = 0, 1, and 2 (*31*). Species diversity was determined solely using samples identified up to the species or species complex level. Samples categorized only at the genus level or those remaining unidentified were excluded from the diversity analyses. The bootstrap iteration count was established at 1000, and the confidence interval level was set at 0.95. A Venn diagram was constructed using R package VennDiagram 1.7.3 (*32*) to assess the overall species overlap between both surveys. Furthermore, the total species count in the market for both surveys was assessed using SpadeR Online (*33*, *34*), using abundance data across six distinct models. These models use the frequencies of rare species to approximate the number of undetected species within each market, along with 95% confidence intervals.

We also assessed the proportion of threatened species present in the identified small fin samples by species and by relative contribution for each of the IUCN Red List categories and presented a direct comparison of the species composition of the most common species (i.e., those with >1% of the identified samples) in both surveys.

The relative contribution of each species found in the small fin survey was plotted against the geographic distribution of each species represented by their presence/absence in each FAO-MFAs to understand the relationships between species relative contribution to international trade, their IUCN Red List status, and their geographic distribution. Nonparametric Spearman and Kendall correlation tests were conducted to assess the correlation between relative contribution and geographic distribution.

In addition, to highlight the regions of the world contributing to the international trade in species with narrow distribution ranges, we mapped the occurrence of each range-restricted species found in the markets within each FAO-MFA, with range-restricted species defined here as those present in seven or fewer FAO-MFAs. We assigned a score to each FAO-MFA to indicate how many species distributions fell within its boundaries and plotted these scores on a map.

## References

[1] C. S. Sherman, C. A. Simpfendorfer, N. Pacoureau, J. H. Matsushiba, H. F. Yan, R. H. L. Walls, C. L. Rigby, W. J. VanderWright, R. W. Jabado, R. A. Pollom, J. K. Carlson, P. Charvet, A. B. Ali, J. Fahmi, D. H. Cheok, K. B. Derrick, B. Herman, T. D. Finucci, M. L. D. Eddy, C. G. Palomares, B. Avalos-Castillo, M.-P. Blanco-Parra Kinattumkara, M. Dharmadi, D. Espinoza, A. B. Fernando, P. A. Haque, A. F. Mejía-Falla, J. C. Navia, J. Pérez-Jiménez, R. R. Utzurrum, N. K. Y. Dulvy, Half a century of rising extinction risk of coral reef sharks and rays. Nat. Commun. 14, 15 (2023). 36650137 10.1038/s41467-022-35091-x PMC9845228

[2] N. Pacoureau, C. L. Rigby, P. M. Kyne, R. B. Sherley, H. Winker, J. K. Carlson, S. V. Fordham, R. Barreto, D. Fernando, M. P. Francis, R. W. Jabado, K. B. Herman, K.-M. Liu, A. D. Marshall, R. A. Pollom, E. V. Romanov, C. A. Simpfendorfer, J. S. Yin, H. K. Kindsvater, N. K. Dulvy, Half a century of global decline in oceanic sharks and rays. Nature 589, 567–571 (2021). 33505035 10.1038/s41586-020-03173-9

[3] D. Cardeñosa, S. K. Shea, H. Zhang, G. A. Fischer, C. A. Simpfendorfer, D. D. Chapman, Two thirds of species in a global shark fin trade hub are threatened with extinction: Conservation potential of international trade regulations for coastal sharks. Conserv. Lett. 15, e12910 (2022).

[4] J. A. Musick, G. Burgess, G. Cailliet, M. Camhi, S. Fordham, Management of Sharks and Their Relatives (Elasmobranchii). Fisheries 25, 9–13 (2000).

[5] S. C. Clarke, J. E. Magnussen, D. L. Abercrombie, M. K. McAllister, M. S. Shivji, Identification of shark species composition and proportion in the Hong Kong shark fin market based on molecular genetics and trade records. Conserv. Biol. 20, 201–211 (2006). 16909673 10.1111/j.1523-1739.2005.00247.x

[6] A. T. Fields, G. A. Fischer, S. K. H. Shea, H. Zhang, D. L. Abercrombie, K. A. Feldheim, E. A. Babcock, D. D. Chapman, Species composition of the international shark fin trade assessed through a retail-market survey in Hong Kong. Conserv. Biol. 32, 376–389 (2018). 29077226 10.1111/cobi.13043

[7] D. Cardeñosa, A. T. Fields, E. A. Babcock, H. Zhang, K. Feldheim, S. K. H. Shea, G. A. Fischer, D. D. Chapman, CITES-listed sharks remain among the top species in the contemporary fin trade. Conserv. Lett. 11, e12457 (2018).

[8] D. Cardeñosa, A. T. Fields, E. A. Babcock, S. K. H. Shea, K. A. Feldheim, D. D. Chapman, Species composition of the largest shark fin retail-market in mainland China. Sci. Rep. 10, 12914 (2020). 32737392 10.1038/s41598-020-69555-1 PMC7395743

[9] D. Cardeñosa, J. Quinlan, K. H. Shea, D. D. Chapman, Multiplex real-time PCR assay to detect illegal trade of CITES-listed shark species. Sci. Rep. 8, 16313 (2018). 30397246 10.1038/s41598-018-34663-6 PMC6218538

[10] D. Abercrombie, D. D. Chapman, S. J. B. Gulak, J. K. Carlson, Visual identification of fins from common elasmobranch in the Northwest Atlantic Ocean. NMFS-SEFSC 643, 48–51 (2013).

[11] D. Cardeñosa, K. H. Shea, H. Zhang, K. Feldheim, G. A. Fischer, D. D. Chapman, Small fins, large trade: A snapshot of the species composition of low-value shark fins in the Hong Kong markets. Anim Conserv 23, 203–211 (2020).

[12] D. Cardeñosa, W. Merten, J. Hyde, Prioritizing global genetic capacity building assistance to implement CITES shark and ray listings. Mar. Policy 106, 103544 (2019).

[13] N. K. Dulvy, N. Pacoureau, C. L. Rigby, R. A. Pollom, R. W. Jabado, D. A. Ebert, B. Finucci, C. M. Pollock, J. Cheok, D. H. Derrick, K. B. Herman, C. S. Sherman, W. J. VanderWright, J. M. Lawson, R. H. L. Walls, J. K. Carlson, P. Charvet, K. K. Bineesh, D. Fernando, G. M. Ralph, J. H. Matsushiba, C. Hilton-Taylor, S. V. Fordham, C. A. Simpfendorfer, Overfishing drives over one-third of all sharks and rays toward a global extinction crisis. Curr. Biol. 31, 4773–4787.e8 (2021). 34492229 10.1016/j.cub.2021.08.062

[14] G. A. Petrossian, Preventing illegal, unreported and unregulated (IUU) fishing: A situational approach. Biol. Conserv. 189, 39–48 (2015).

[15] W. T. White, C. Charles, P. M. Kyne, M. Harris, Lost before found: A new species of whaler shark Carcharhinus obsolerus from the Western Central Pacific known only from historic records. PLOS ONE 14, e0209387–e0209324 (2019). 30601867 10.1371/journal.pone.0209387 PMC6314596

[16] C. Pimiento, C. Albouy, D. Silvestro, T. L. Mouton, L. Velez, D. Mouillot, A. B. Judah, J. N. Griffin, F. Leprieur, Functional diversity of sharks and rays is highly vulnerable and supported by unique species and locations worldwide. Nat. Commun. 14, 7691 (2023). 38001077 10.1038/s41467-023-43212-3 PMC10673927

[17] F. Dent, S. Clarke, State of the global market for shark products (FAO Fisheries and Aquaculture, Technical Paper No. 590), pp. 187 (2015).

[18] P. M. Kyne, M. R. Heupel, W. T. White, C. A. Simpfendorfer, The Action Plan for Australian Sharks and Rays (National Environmental Science Program, Marine Biodiversity Hub, 2021).

[19] F. Ferretti, R. A. Myers, F. Serena, H. K. Lotze, Loss of large predatory sharks from the Mediterranean Sea. Conserv. Biol. 22, 952–964 (2008). 18544092 10.1111/j.1523-1739.2008.00938.x

[20] R. Barreto, F. Ferretti, J. M. Flemming, A. Amorim, H. Andrade, B. Worm, R. Lessa, Trends in the exploitation of South Atlantic shark populations. Conserv. Biol. 30, 792–804 (2016). 26634410 10.1111/cobi.12663

[21] A. P. Prasetyo, M. Cusa, J. M. Murray, F. Agung, E. Muttaqin, S. Mariani, A. D. McDevitt, Universal closed-tube barcoding for monitoring the shark and ray trade in megadiverse conservation hotspots. iScience 26, 107065 (2023). 37389182 10.1016/j.isci.2023.107065 PMC10300358

[22] J. K. Carlson, L. F. Hale, A. Morgan, G. Burgess, Relative abundance and size of coastal sharks derived from commercial shark longline catch and effort data. J. Fish Biol. 80, 1749–1764 (2012). 22497406 10.1111/j.1095-8649.2011.03193.x

[23] M. R. Heupel, J. K. Carlson, C. A. Simpfendorfer, Shark nursery areas: concepts, definition, characterization and assumptions. Mar. Ecol. Prog. Ser. 337, 287–297 (2007).

[24] S. M. Evans, C. McKenna, S. D. Simpson, J. Tournois, M. J. Genner, Patterns of species range evolution in Indo-Pacific reef assemblages reveal the Coral Triangle as a net source of transoceanic diversity. Biol. Lett. 12, 20160090 (2016). 27330168 10.1098/rsbl.2016.0090 PMC4938039

[25] M. J. Kinney, C. A. Simpfendorfer, Reassessing the value of nursery areas to shark conservation and management. Conserv. Lett. 2, 53–60 (2009).

[26] J. R. Quinlan, S. J. O’Leary, A. T. Fields, M. Benavides, E. Stumpf, R. Carcamo, J. Cruz, D. Lewis, B. Wade, G. Amato, S. Kolokotronis, G. M. Clementi, D. D. Chapman, Using fisher-contributed secondary fins to fill critical shark-fisheries data gaps. Conserv. Biol. 35, 991–1001 (2021). 33538362 10.1111/cobi.13688

[27] G. W.-C. But, H.-Y. Wu, K.-T. Shao, P.-C. Shaw, Rapid detection of CITES-listed shark fin species by loop-mediated isothermal amplification assay with potential for field use. Sci. Rep. 10, 4455 (2020). 32157111 10.1038/s41598-020-61150-8 PMC7064571

[28] M. A. MacNeil, D. D. Chapman, M. Heupel, C. A. Simpfendorfer, M. Heithaus, M. Meekan, E. Harvey, J. Goetze, J. Kiszka, M. E. Bond, L. M. Currey-Randall, C. W. Speed, C. S. Sherman, M. J. Rees, V. Udyawer, K. I. Flowers, G. Clementi, J. Valentin-Albanese, T. Gorham, M. S. Adam, K. Ali, F. Pina-Amargós, J. A. Angulo-Valdés, J. Asher, L. G. Barcia, O. Beaufort, C. Benjamin, A. T. F. Bernard, M. L. Berumen, S. Bierwagen, E. Bonnema, R. M. K. Bown, D. Bradley, E. Brooks, J. J. Brown, D. Buddo, P. Burke, C. Cáceres, D. Cardeñosa, J. C. Carrier, J. E. Caselle, V. Charloo, T. Claverie, E. Clua, J. E. M. Cochran, N. Cook, J. Cramp, B. D’Alberto, M. de Graaf, M. Dornhege, A. Estep, L. Fanovich, N. F. Farabaugh, D. Fernando, A. L. Flam, C. Floros, V. Fourqurean, R. Garla, K. Gastrich, L. George, R. Graham, T. Guttridge, R. S. Hardenstine, S. Heck, A. C. Henderson, H. Hertler, R. Hueter, M. Johnson, S. Jupiter, D. Kasana, S. T. Kessel, B. Kiilu, T. Kirata, B. Kuguru, F. Kyne, T. Langlois, E. J. I. Lédée, S. Lindfield, A. Luna-Acosta, J. Maggs, B. M. Manjaji-Matsumoto, A. Marshall, P. Matich, E. M. Combs, D. M. Lean, L. Meggs, S. Moore, S. Mukherji, R. Murray, M. Kaimuddin, S. J. Newman, J. Nogués, C. Obota, O. O. Shea, K. Osuka, Y. P. Papastamatiou, N. Perera, B. Peterson, A. Ponzo, A. Prasetyo, L. M. S. Quamar, J. Quinlan, A. Ruiz-Abierno, E. Sala, M. Samoilys, M. Schärer-Umpierre, A. Schlaff, N. Simpson, A. N. H. Smith, L. Sparks, A. Tanna, R. Torres, M. J. Travers, M. van Zinnicq Bergmann, L. Vigliola, J. Ward, A. M. Watts, C. Wen, E. Whitman, A. J. Wirsing, A. Wothke, E. Zarza-Gonzâlez, J. E. Cinner, Global status and conservation potential of reef sharks. Nature 583, 801–806 (2020). 32699418 10.1038/s41586-020-2519-y

[29] D. Cardeñosa, A. Fields, D. Abercrombie, K. Feldheim, S. K. H. Shea, D. D. Chapman, A multiplex PCR mini-barcode assay to identify processed shark products in the global trade. PLOS ONE 12, e0185368–e0185369 (2017). 29020095 10.1371/journal.pone.0185368 PMC5636071

[30] A. Chao, K. H. Ma, T. C. Hsieh, iNEXT (iNterpolation and EXTrapolation) Online: Software for Interpolation and Extrapolation of Species Diversity. Program and User’s Guide (2016); https://chao.shinyapps.io/iNEXTOnline/.

[31] A. Chao, N. J. Gotelli, T. C. Hsieh, E. L. Sander, K. H. Ma, R. K. Colwell, A. M. Ellison, Rarefaction and extrapolation with Hill numbers: a framework for sampling and estimation in species diversity studies. Ecological monographs 84, 45–67 (2014).

[32] H. Chen, VennDiagram: Generate High-Resolution Venn and Euler Plots. R package version 1.7.3 (2022); http://CRAN.R-project.org/package=VennDiagram.

[33] A. Chao, K. H. Ma, T. C. Hsieh, C.-H. Chiu, Online Program SpadeR (Species-richness Prediction And Diversity Estimation in R). Program and User’s Guide published (2015); http://chao.stat.nthu.edu.tw/wordpress/software_download/.

[34] A. Chao, K. H. Ma, T. C. Hsieh, C.-H. Chiu, SpadeR: Species prediction and diversity estimation with R. R package version 0.1.1. (2016).

[35] A. Chao, Nonparametric Estimation of the Number of the Classes in a Population. Scand. J. Statist. 11, 265–270 (1984).

[36] C.-H. Chiu, L. Jost, A. Chao, Phylogenetic beta diversity, similarity, and differentiation measures based on Hill numbers. Ecological Monographs 84, 21–44 (2014); 10.1890/12-0960.1.

[37] Chao, A., & Lee, S.-M., Estimating the Number of Classes via Sample Coverage. J. Am. Stat. Assoc. 87, 210–217 (1991); https://doi.org/10.1080/01621459.1992.10475194.

